# Effects of Magnetic Biochar Addition on Mesophilic Anaerobic Digestion of Sewage Sludge

**DOI:** 10.3390/ijerph20054278

**Published:** 2023-02-28

**Authors:** Li Jiang, Yanru Zhang, Yi Zhu, Zhongliang Huang, Jing Huang, Zijian Wu, Xuan Zhang, Xiaoli Qin, Hui Li

**Affiliations:** 1 College of Resources and Environment, Yangtze University, Wuhan 430100, China; 2 State Key Laboratory of Utilization of Woody Oil Resource, Hunan Academy of Forestry, Changsha 410004, China

**Keywords:** magnetic biochar, anaerobic digestion, kinetic model, sewage sludge, biogas yield

## Abstract

As a low-cost additive to anaerobic digestion (AD), magnetic biochar (MBC) can act as an electron conductor to promote electron transfer to enhance biogas production performance in the AD process of sewage sludge and has thus attracted much attention in research and industrial applications. In the present work, *Camellia oleifera shell* (COS) was used to produce MBC as an additive for mesophilic AD of sewage sludge, in order to explore the effect of MBC on the mesophilic AD process and its enhancement mechanism. Analysis by scanning electron microscopy (SEM), energy dispersive X-ray spectroscopy (EDS), Fourier-transform infrared spectrometry (FTIR), and X-ray diffraction (XRD) further confirmed that biochar was successfully magnetized. The yield of biogas from sewage sludge was enhanced by 14.68–39.24% with the addition of MBC, and the removal efficiency of total solid (TS), volatile solids (VS), and soluble chemical oxygen demand (sCOD) were 28.99–46.13%, 32.22–48.62%, and 84.18–86.71%, respectively. According to the Modified Gompertz Model and Cone Model, the optimum dosage of MBC was 20 mg/g TS. The maximum methane production rate (R_m_) was 15.58% higher than that of the control reactor, while the lag-phase (λ) was 43.78% shorter than the control group. The concentration of soluble Fe^2+^ and Fe^3+^ were also detected in this study to analyze the function of MBC for improving biogas production performance from sewage sludge. The biogas production was increased when soluble Fe^3+^ was reduced to soluble Fe^2+^. Overall, the MBC was beneficial to the resource utilization of COS and showed a good prospect for improving mesophilic AD performance.

## 1. Introduction

Large amounts of sewage sludge are generated by sewage treatment plants, and a great quantity of pathogens, organic pollutants, antibiotic resistance genes, etc., in sewage sludge will result in serious pollution problems [1,2]. Therefore, the proper treatment technology for sewage sludge is essential. So far, anaerobic digestion (AD) is a relatively stable and extensively applied technology for treating sewage sludge, food wastes, and animal manure [3,4]. AD includes four stages: hydrolysis, acidification, acetogenesis, and methanogenesis [5]. Waste can be first decomposed into simple organic compounds and these simple organic compounds are further acidified into volatile fatty acids (VFAs), which are converted into biogas during the acetogenesis and methanogenesis stages [6,7]. However, AD performance often suffers influence from factors such as the low degradation rate of organic matter, the restrictive hydrolysis–acidification step, and process conditions. To improve the performance of AD, scholars have proposed some improvements, including pre-treatment, adding additive, and co-digestion strategy, etc., but adding additive is simpler in operation than co-digestion, and superior to pre-treatment in cost and energy consumption [8].

Using additives is a widely applied improvement, and biochar has been the dominant additive [9,10]. Recently, using biochar materials prepared from agroforestry waste have gained attention. *Camellia oleifera* shell (COS) is a by-product of the *Camellia oleifera* Abel. (*Camellia L.*) industry, and is used as a solid fuel or discarded in the environment, leading to potential environmental pollution and significant waste of resources. COS is rich in lignin and cellulose, which can be prepared as biochar (labeled as C-BC) with environmental and economic benefits. Biochar can facilitate direct interspecies electron transfer (DIET) as a conductive material, and enhance biogas production [11]. Moreover, biochar has certain adsorption properties due to its rich pore structure and high cation exchange capacity [12]. To achieve stability of the AD system, pH adjustment often becomes necessary, and biochar can buffer the effect of organic acids [13]. Adding biochar during AD has been reported positive benefits; Zhang et al., found that the addition of biochar reduces the inhibition of anaerobic digestion by pollutants and promotes archaea growth and biogas production [14]. Kizito et al., also reported that biochar reduced the lag phase to 4–5.4 d and the cumulative methane increased by 37–56% [13].

Pyrolysis temperature has effects on the physicochemical properties of biochar. A series of treatments have been tried to improve the performance of biochar. Magnetization of biochar is a common modification technique, and biochar with magnetism has new advantages in practical applications. As reported, magnetite has a positive impact on facilitating interspecies electron transfer (IET) among anaerobic microorganisms [15]. On one hand, magnetite can establish DIET between syntrophic bacteria and methanogens to replace contact between conductive pili and cells [16]. On the other hand, magnetite can enrich iron-reducing bacteria to participate in DIET, and iron-reducing bacteria can also decompose complex organic matter by reducing dissimilatory iron and accelerating methane production [17]. Therefore, to enhance the adsorption properties and electroactive properties of C-BC during AD, magnetic modification of biochar is possible. Generally, an iron ion solution is used to impregnate biomass, introduce iron ions into biomass, and convert them into magnetic iron oxides through pyrolysis to obtain magnetic biochar (MBC).

Furthermore, FeCl_3_ is commonly used as a flocculant in wastewater treatment plants and has been widely proven to increase biogas production. Yu et al., demonstrated that FeCl_3_ can enrich *Coprothermobacter* for protein fermentation and *Methanosarcina* to increase biogas production [18]. Scholars also found that FeCl_3_ can buffer pH, provide electrons and reduce the inhibition of H_2_S on microorganisms, and significantly promotes biogas production [19,20]. Therefore, we used an FeCl_3_ solution and COS to prepare MBC in this study.

The biogas production kinetic model can be useful to investigate the effect of magnetic biochar addition on AD performance, which is important for predicting and optimizing AD. Biochemical methane potential is the maximum amount of methane that can be produced from a feedstock under certain AD conditions [21]. Scholars have proposed various kinetic models to predict methane production potential, and the most suitable model is the Modified Gompertz Model. The Cone Model is another gas production kinetic model. El-Mashad et al., reported that the Cone Model is the best fit in the co-digestion experiments with the switchgrass and spirulina platensis algae [22].

The purpose of this research is to investigate the effects of adding different dosages of MBC to AD under mesophilic conditions. Specifically, this research has three key goals: (1) to determine the performance of AD under mesophilic conditions with MBC at different dosages. This will mainly analyze the changes in the parameters including biogas yield, organic matter removal efficiency, soluble protein, soluble polysaccharide, and VFAs; (2) to monitor the change trend of Fe^3+^/Fe^2+^ throughout the mesophilic AD cycle; (3) to evaluate the effect of MBC addition on kinetic parameters and predict the biogas production potential of AD.

## 2. Materials and Methods

### 2.1. Preparation of MBC

The raw common COS was collected, washed, dried, and crushed to pass through a 0.125–0.154 mm sieve. First, the prepared COS biomass was immersed in KOH solution, heated and stirred for 2 h, and then washed and dried after cooling. Subsequently, the COS biomass was added to the FeCl_3_ solution, heated, stirred, and washed until pH was constant, then washed, dried, and then pyrolyzed to obtain MBC [23]. KOH and FeCl_3_ (97.0% purity) were purchased from Macklin biochemical technology Co., Ltd., Shanghai, China.

### 2.2. Sewage Sludge Samples

The seed sludge and dewatered sludge were both collected from a municipal wastewater treatment plant in Changsha, China. The seed sludge was filtered through a 2 mm mesh screen after collection and kept in inert nitrogen atmosphere for 30 min, and then placed in a constant temperature incubator at 35 °C to enrich anaerobic microorganisms. The feed sludge was a mixture of dewatered sludge and deionized water in a certain proportion. The main physicochemical characteristics of sludge were analyzed in triplicate, and the results are shown in Table 1.

### 2.3. Batch Experimental Setup

Batch experiments were conducted with 12 glass reactors, each with a working volume of 3 L, the mixture of seed sludge and feed sludge with a ratio of 2:1 was added into the reactors and then filled with an inert nitrogen atmosphere for 15 min. The batch experiment was conducted in a constant temperature water bath at 35.0 ± 1 °C for 41 days. The dosages of MBC were set at 5 mg/g TS (MB_1), 10 mg/g TS (MB_2), 20 mg/g TS (MB_3), 40 mg/g TS (MB_4), and 80 mg/g TS (MB_5). The control reactor was set without MBC. Biogas volume was measured regularly and anaerobically digested samples were collected until biogas production stopped. Each reactor was tested in triplicate.

### 2.4. Analytical Methods

The surface structure and composition of MBC were detected by scanning electron microscopy (SEM). Energy dispersive X-ray spectroscopy (EDS) was used to examine surface elemental composition and distribution. The functional groups analysis was identified by Fourier-transform infrared spectrometer (FTIR). X-ray diffractometer (XRD) was carried out to identify chemical composition.

The daily biogas production of the batch experiment was determined by the saturated salt water-replace method. The pH of each sample was measured by pH meter (Mettler Toledo, Zaventem, Belgium). For measurements of TS and VS of sewage sludge, the sewage sludge was placed into a constant temperature drying oven for drying at 105 °C, and then placed in a muffle furnace at 600 °C for 2 h. The TS and VS were measured by the differential weight method. The sCOD was determined by the potassium dichromate digestion method, the concentration of soluble protein was determined by ultraviolet spectrophotometry using bovine serum protein as standard, and the concentration of soluble polysaccharides was determined by the phenol–sulfuric acid method [4,24]. The concentrations of VFAs (mainly acetic acid, propionic acid, n-butyric acid, iso-butyric acid, n-valeric acid, and iso-valeric acid) were measured using a gas chromatograph equipped with a flame ionization detector (Agilent 7890A, Santa Clara, CA, USA). The detailed analytical methods of these physicochemical parameters have been described in the literature [25,26]. The concentration of soluble Fe^3+^ and soluble Fe^2+^ were measured using the o-phenanthroline spectrophotometric method. o-phenanthroline is a chromogenic reagent that reacts with Fe^2+^ to produce a red complex [27]. The soluble fraction was obtained by centrifuging samples at 8000 rpm/min. The absorbance of Fe^2+^ at 510 nm was determined by a spectrophotometric system after o-phenanthroline was added. Subsequently, in order to determine the total iron concentration, hydroxylamine hydrochloride and o-phenanthroline were added to partial samples to reduce Fe^3+^ to Fe^2+^ and the Fe^3+^ concentration was the difference between the total iron concentration and Fe^2+^ concentration. Three parallel samples were conducted for each experiment.

### 2.5. Kinetic Models

Two kinetic models, the Modified Gompertz Model and Cone Model, were chosen to describe the influence on kinetic parameters of the AD system with different dosages of MBC [21,28]. These model equations and parameters are shown in Table 2. The statistical analysis of data, kinetic models, and draw plot were conducted by Origin 2021 (https://www.originlab.com/, accessed on 2 January 2023).

## 3. Results and Discussion

### 3.1. Characterization of MBC

**Figure 1:**
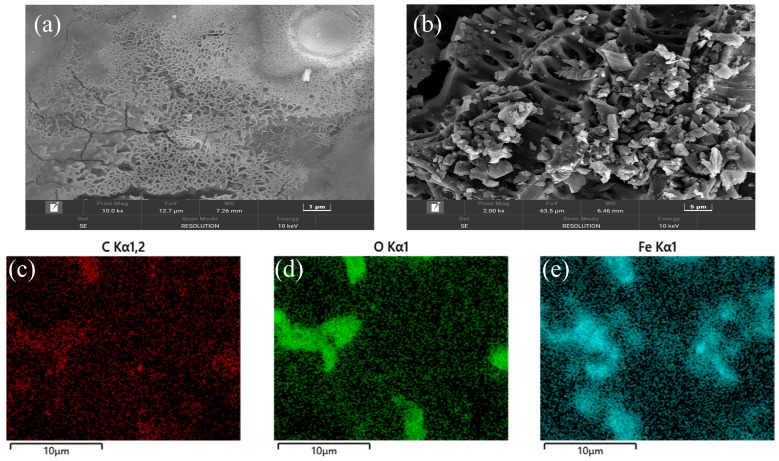
SEM images of MBC (**a**) and C-BC (**b**). EDS mapping image of MBC (**c**–**e**).

The effect of biochar on DIET is mainly reflected in the electron transport capacity, the stronger the electron transport capacity, the higher the efficiency of biochar-mediated DIET. In the SEM images of MBC and C-BC (Figure 1a,b), the microporous structure of MBC was more intensive than that of C-BC and provided a conducive environment for microbial attachment. The EDS analysis (Figure 1c–e) showed that the surface of MBC contained C, O, and Fe. In addition, as with the magnetized leaf biochar studied by Huang et al. [29], MBC had a smaller surface area and pore volume than C-BC, probably due to the iron oxide crystals formed by magnetization that blocked the original pores of C-BC.

**Figure 2:**
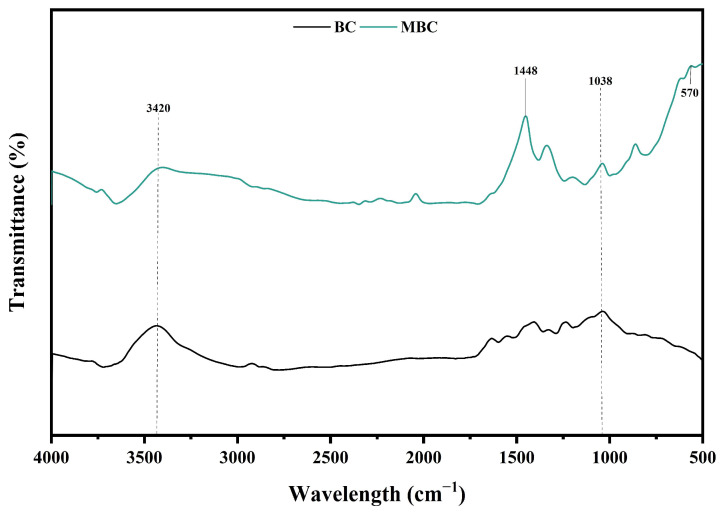
FTIR spectra of MBC and C-BC.

The FTIR spectra showed the changes of biochar functional groups before and after magnetization (Figure 2). Both C-BC and MBC had obvious absorption peaks for O-H stretching vibration at 3420 cm^−1^, which was conducive to the occurrence of ion exchange [30]. The peak at approximately 1448 cm^−1^ was assigned to C=C stretching vibration of the aromatic structure and only appeared in MBC, indicating that MBC has high aromaticity and stability, and the aromatic structure can be used as an electron donor [31]. The peak at 1038 cm^−1^ belonged to C-O bond of the ether, and MBC peak intensity increased compared to C-BC, indicating an increase in oxygen-containing functional groups [32]. The FTIR spectra of MBC showed a new peak at 570 cm^−1^, corresponding to the Fe-O characteristic peak, which indicated that FeCl_3_ was successfully loaded on biochar [33].

**Figure 3:**
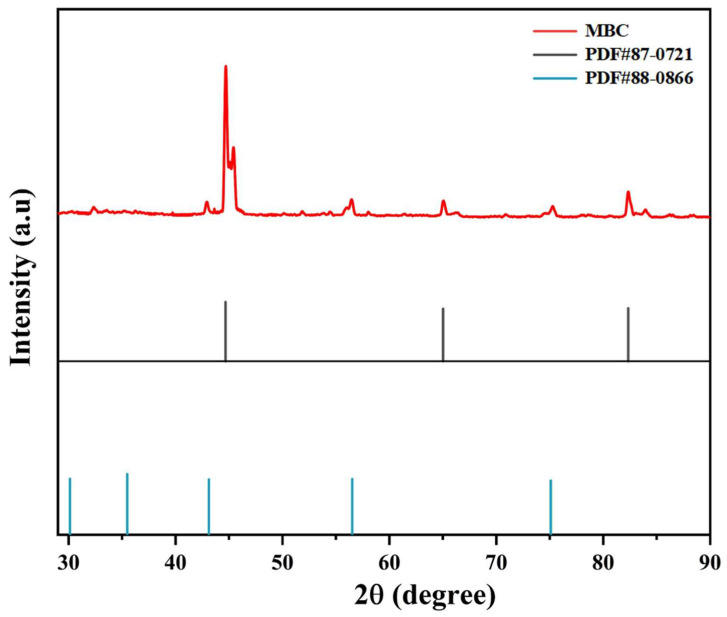
XRD pattern of MBC.

Figure 3 showed the XRD pattern of MBC, which confirmed the main chemical composition of the material. The major crystalline phases of MBC were magnetite and Fe, further confirming the successful magnetization of biochar. The diffraction peaks of magnetite were 2θ = 30.12°, 35.48°, 43.12°, 57.03°, and 71.04°, which was consistent with the standard card of magnetite (PDF#88-0866). Simultaneously, three new characteristic peaks at 2θ = 44.64°, 65.07°, and 82.34° were observed, which corresponded to the standard card of Fe (PDF#87-0721). MBC may have high electron conductivity due to the contents of magnetite and Fe.

### 3.2. Cumulative Biogas Yield

**Figure 4:**
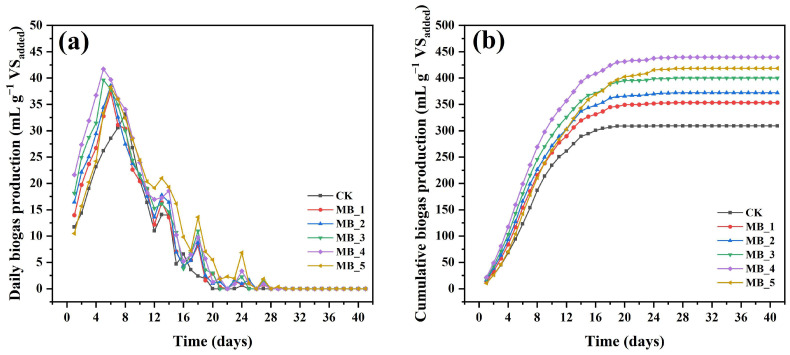
Daily biogas production (**a**) and cumulative biogas production (**b**).

In order to investigate the effect of MBC on the biogas production process of mesophilic AD, five different doses of MBC were added to the anaerobic system and compared with the control reactor without addition. Daily biogas production with MBC addition was within the range of 0–41.71 mL g^−1^ VS_added_ (Figure 4a). From the fifth day, the biogas production of the MBC reactors gradually reached the peak, and peak daily biogas production was followed the order of MB_4 (41.71 mL g^−1^ VS_added_) > MB_3 (39.64 mL g^−1^ VS_added_) > MB_2 (38.57 mL g^−1^ VS_added_) > MB_5 (38.30 mL g^−1^ VS_added_) > MB_1 (37.26 mL g^−1^ VS_added_). At about the eighth day, the control reactor slowly reached the peak daily biogas production (33.17 mL g^−1^ VS_added_), which was lower than all reactors with MBC addition. The results confirmed that MBC resulted in earlier peaks of daily biogas production.

The cumulative biogas production of six reactors is shown in Figure 4b. After 41 days of digestion experiments, the cumulative gas production of the six reactors was 309.20 mL g^−1^ VS_added_ (CK), 353.58 mL g^−1^ VS_added_ (MB_1), 372.28 mL g^−1^ VS_added_ (MB_2), 400.16 mL g^−1^ VS_added_ (MB_3), 439.71 mL g^−1^ VS_added_ (MB_4), and 418.54 mL g^−1^ VS_added_ (MB_5). The cumulative biogas production was increased from 14.35% to 42.21% with the increase of MBC dosage, and the order of the enhancement efficiency in final biogas production was MB_1 (14.35%) < MB_2 (20.40%) < MB_3 (29.42%) < MB_5 (35.36%) < MB_4 (42.21%). The results indicated that 40 mg/g TS (MB_4) was the optimum dosage in this study.

Compared with the CK reactor, MBC addition had a facilitating effect on biogas production, indicating that MBC promoted AD. MBC could promote the formation of electroactive bacteria, and not only realize long-distance interspecific electron exchange, but also facilitate microbial adsorption and fixation, form clusters or biofilms, and indirectly mediate the occurrence of DIET, which was beneficial for AD [34,35]. Furthermore, MBC can actively stimulate the activity of enzymes in metabolic pathways during the peak of biogas production [36].

In the five experimental groups with different dosages, MB_5 (80 mg/g TS) was less efficient than MB_4 (40 mg/g TS) in enhancing biogas production under the medium temperature condition, and a significant lag phase was observed before the fourth day. These effects suggested that excessive addition of MBC posed a potential risk to methane production. This result was consistent with earlier studies. For example, Yu et al., reported that an appropriate dosage of Fe^3+^ may help to inhibit the accumulation of VFAs, while elevated concentration may lead to systemic breakdown [37]. Ali et al., also found that the maximum biomethane production can be obtained at a dosage of 0.6 g/L when the dosages of Fe^3+^ were set at 0.025–5.4 g/L [38]. In addition, the literature has shown that the low methane production may also be related to the adsorption of MBC to carbon sources [39].

### 3.3. Effects of MBC on AD Performance

#### 3.3.1. Removal of Organic Matter

**Figure 5:**
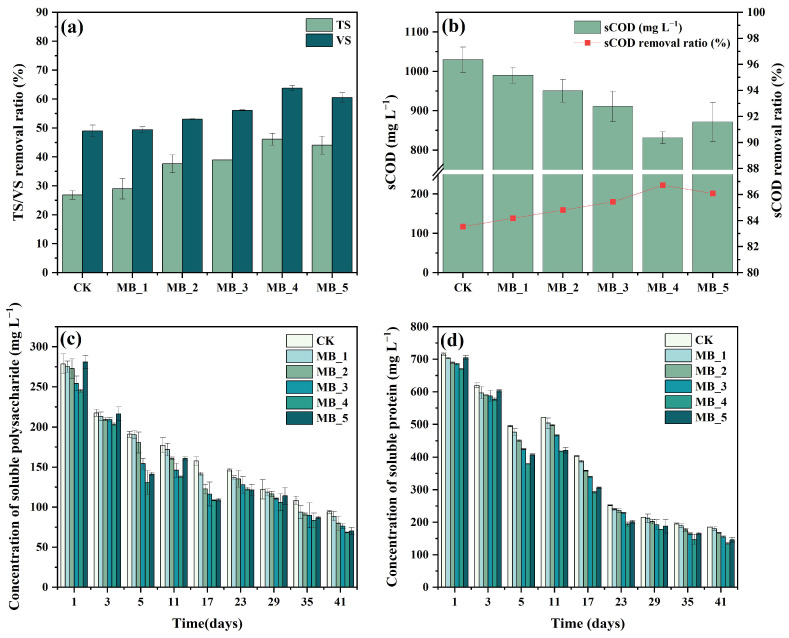
Effect of MBC on AD performance for 41 days. TS/VS removal efficiency (**a**). sCOD content and removal efficiency (**b**). Changes of soluble polysaccharide (**c**) and soluble protein (**d**).

Besides the biogas production index, the removal ratio of TS, VS, and sCOD were also significant parameters for the performance of AD [40]. Variations of TS and VS are presented in Figure 5a. The TS removal efficiency of six experimental groups was 26.86% (CK), 28.99% (MB_1), 37.65% (MB_2), 38.94% (MB_3), 46.13% (MB_4), and 44.06% (MB_5), respectively. The removal efficiency of VS follows the same rule as TS, and was 48.99% (CK),49.42% (MB_1), 53.05% (MB_2), 56.09% (MB_3), 63.81% (MB_4), and 60.58% (MB_5). Compared with the CK reactor, the ultimate removal efficiency of TS increased by 71.74% in MB_4 and the VS removal efficiency of MB_4 increased by 30.25%. The higher VS removal efficiency indicated the enhancement of solid organic matter hydrolysis with MBC. In general, the addition of MBC can effectively improve the removal efficiencies of TS and VS, the removal efficiency increases with the increase of dosage, and the inhibition was observed at 80 mg/g TS (MB_5).

The content and removal rate of sCOD in different reactors can be seen from Figure 5b. Overall, the sCOD concentrations were decreased by 83.54%, 84.18%, 84.81%, 85.44%, 86.71%, and 86.08% with the MBC dosages of 0 mg/g TS, 5 mg/g TS, 10 mg/g TS, 20 mg/g TS, 40 mg/g TS, and 80 mg/g TS, respectively. Compared with the other reactors, the addition of 40 mg/g TS MBC had the best removal of sCOD, while adding 80 mg/g TS MBC inhibited the degradation of sCOD. The final degradation rate of sCOD was limited by the substrate concentration and the organic matter was fully decomposed and utilized.

The variation trend of sCOD removal efficiency was similar to the trend of TS/VS. It indicated that the appropriate concentrations of MBC could accelerate the decomposition of organic matter. MBC could promote the oxidation–reduction process in the digestive system through DIET and accelerate the degradation of organic matter. The reduction of organic matter would promote biogas production at the methanogenic stage, showing that the addition of MBC improved the degradation of organic matter and enhanced biogas production.

#### 3.3.2. Change of Soluble Protein and Soluble Polysaccharide

In order to further explore the effect of MBC on the hydrolysis of AD, soluble protein and soluble polysaccharide were the important parameters to react to the change of organic matter in the hydrolysis stage [28,41]. Figure 5c,d shows the concentration changes of soluble polysaccharide and soluble protein in six reactors during 41 days of the experiment. It could be seen that the soluble polysaccharide concentrations of all reactors were decreased, by 77.22%, 78.60%, 80.66%, 81.54%, 83.43%, and 83.02%, when the MBC addition dosages were 0 mg/g TS, 5 mg/g TS, 10 mg/g TS, 20 mg/g TS, 40 mg/g TS, and 80 mg/g TS, respectively.

Sludge contains a large amount of protein, which is an important nitrogen source for microorganism growth [42]. The soluble protein removal efficiency of all the experimental groups was 59.12% (CK), 60.24% (MB_1), 63.08% (MB_2), 65.69% (MB_3), 70.36% (MB_4), and 67.74% (MB_5), respectively. Among them, the addition of 40 mg/g TS MBC had the fastest rate to degrade soluble protein; it indicated that the soluble protein had been rapidly hydrolyzed and acidified into amino acids [43].

Based on the results, the hydrolysis of soluble protein and soluble polysaccharide was similar to the removal trend of organic matter. Protease and cellulase are the key enzymes to hydrolyze soluble polysaccharides and soluble proteins [44]. When the dose of MBC reached 80 mg/g TS, the activities of protease and cellulase would be inhibited, which leads to the low degradation rate of soluble protein and soluble polysaccharide and the decrease of methane production.

#### 3.3.3. Change of VFAs and pH

VFAs and pH are important indexes to judge the stability of the AD system [45]. The pH value has great influence on microbial activity in the AD system, and the optimal pH range for stable operation of the digestive system is 6.8–7.2 [46]. In this study, the pH values of all experimental groups were within the range of 6.93–7.46, and the whole AD process was relatively stable. There is no acidified phenomenon in all treatments.

**Figure 6:**
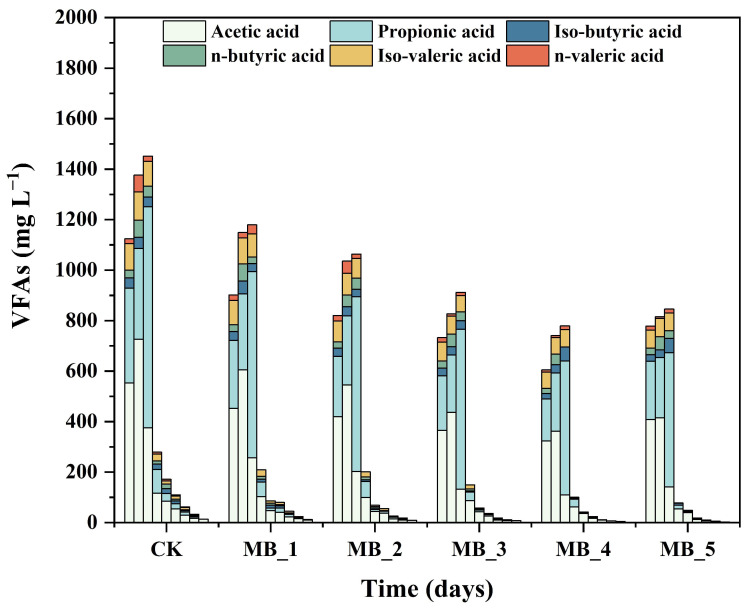
Variation of VFAs during mesophilic AD.

Variation in the VFAs during AD is shown in Figure 6. During the process of hydrolysis and acidification, soluble organic matter was decomposed into VFAs, which were further converted into methane [47]. VFAs mainly include acetic acid, propionic acid, butyric acid, and valeric acid. From Figure 6, the VEA concentrations of six reactors first increased and then decreased. The peak values of VFA concentrations appeared around the fifth day. The VFA concentrations decreased sharply from days 5 to 11, and then remained at lower concentrations. In each group, acetic acid and propionic acid were the main metabolites. All groups with MBC showed lower acetic acid concentrations than the CK group because the addition of magnetic carbon-based materials increased the electron shuttling efficiency [48].

During the stage of hydrolysis and acid production, complex biodegradable organic matter was decomposed into soluble low molecular weight organic substances, which were under the action of protease and cellulase [49]. Furthermore, low molecular weight organic substances were decomposed into small molecular substances through complex biochemical metabolic reactions, including acetic acid, propionic acid, butyric acid, valerate acid, hydrogen, carbon dioxide, and so on. Compared with the change trends of VFAs, soluble protein, and soluble polysaccharide, it can be seen that the concentration of VFAs gradually increased when the concentration of soluble protein and soluble polysaccharide decreased in the first 5 days of AD. After the fifth day, when the concentration of VFAs decreased, the concentration of soluble protein and soluble polysaccharide increased. Finally, the concentration of VFAs was further decreased, as well as soluble proteins and soluble polysaccharide [50].

### 3.4. Kinetic Analyses

Kinetic models can be used to predict the biomethane production performance in AD and can be helpful to develop equations describing the complex biogas production behavior in the digestive system. AD is a dynamic system, and it is meaningful to establish mathematical expressions for various biochemical reaction parameters in the AD process. Therefore, it is very important to select appropriate kinetic models to analyze the cumulative biogas production in the digestive system after adding MBC.

In this study, the Modified Gompertz Model and Cone Model were used for the kinetic analysis to study the cumulative biogas production of six reactors. The results of the kinetic analysis are shown in Table 3 and Table 4. The correlation coefficient (R^2^) of the Modified Gompertz Model and the Cone Model were in the range of 0.9988–0.9997 and 0.9941–0.9971, respectively. The R^2^ obtained by the Modified Gompertz Model was higher than that of the Cone Model. What is more, in agreement with the result of R^2^, the difference (the calculated deviation between the predicted and measured values, which was close to or <10%) obtained from the Modified Gompertz Model was lower than the Cone Model for the AD of six reactors, the prediction value obtained by the Modified Gompertz Model was closer to the measured value. These results indicated that the Modified Gompertz Model was better than the Cone Model to fit the biogas production process of sludge AD after adding MBC.

*B*_0_ is defined as the ultimate cumulative biogas production. *B*_0_ obtained by the Cone Model and Modified Gompertz Model followed the same order: MB_4 > MB_5 > MB_3 > MB_2 > MB_1 > CK. The order of *B*_0_ was consistent with the experimental results. The maximum biogas production rate (*R_m_*) can be used to evaluate the biogas production of different digestion reaction systems. As shown in Table 3, the *R_m_* of six reactors was 32.23 mL g^−1^ VS d^−1^ (CK), 33.37 mL g^−1^ VS d^−1^ (MB_1), 34.24 mL g^−1^ VS d^−1^ (MB_2), 36.10 mL g^−1^ vs. d^−1^ (MB_3), 39.18 mL g^−1^ vs. d^−1^ (MB_4), and 33.66 mL g^−1^ vs. d^−1^ (MB_5), respectively. *R_m_* in the MB_4 reactor was increased by 21.56% more than the CK reactor, demonstrating that the appropriate addition of MBC has a positive impact on biogas production.

λ refers to the lag phase time, which indicated the time required for microorganisms in AD system to adapt to a new environment, and the lower the value of *λ*, the faster the digestion process starts [51]. In this study, *λ* values were found in the ranges of 2.01 d (CK), 1.46 d (MB_1), 1.27 d (MB_2), 1.11 d (MB_3), 1.02 d (MB_4), and 1.91 d (MB_5), respectively. The result indicated that MBC has favorable electrical conductivity, so leads to the reactors with MBC added having shorter lag phase time than those without MBC added. When the dosage of MBC was excessive, it would inhibit the activities of methanogens and related enzymes, and then prolong the lag phase time.

A higher value of *K*, which is defined as the hydrolysis rate constant, indicates a faster hydrolysis rate during AD [52]. As shown in Table 4, from the results of the Cone Model analysis, the *K* values of all experimental groups were 0.1470 d^−1^ (CK), 0.1491 d^−1^ (MB_1), 0.1504 d^−1^ (MB_2), 0.1517 d^−1^ (MB_3), 0.1522 d^−1^ (MB_4), and 0.1227 d^−1^ (MB_5), respectively. Compared with the CK reactor, the *K* value for MBC at dosages ranging from 5 mg/g TS to 40 mg/g TS increased by 0.0020 d^−1^ to 0.0052 d^−1^. However, when the dosage of MBC was set at 80 mg/g TS, the *K* value decreased by 0.0243 d^−1^ compared with the CK group. These results apparently demonstrated that the dosage of MBC should be moderate, and excessive dosage would limit the hydrolysis process.

### 3.5. Iron Concentration

**Figure 7:**
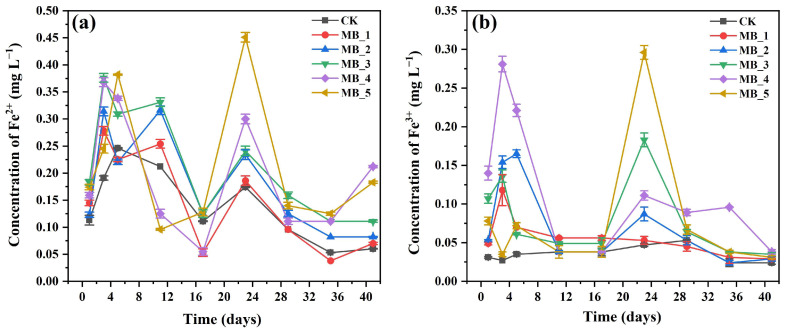
The concentration variations of Fe^3+^ (**a**) and Fe^2+^ (**b**).

Iron mainly exists in the form of soluble Fe^3+^ and Fe^2+^ in the AD process of sewage sludge. Figure 7 shows the concentration variations of Fe^2+^ and Fe^3+^ in the six reactors. FeCl_3_ was a flocculant commonly used in wastewater treatment plants, thus the control reactor also had a small number of iron ions, and a concentration of soluble Fe^3+^ with a small change (0.024–0.053 mg/L). In the early stage of AD, the concentration of Fe^3+^ increased rapidly, due to the Fe^3+^ loaded on the MBC being released. During the hydrogen production and acid production phases, the concentration of Fe^3+^ decreased rapidly. The large amounts of reduced state hydrogen were produced to reduce Fe^3+^ to Fe^2+^ and provided electrons to the digestive system. It was also enriched with iron-reducing bacteria, which can promote the reduction of dissimilatory iron [53]. In addition, the presence of MBC ensured a continuous supply of iron ions during the AD process. On the 23rd day, the concentration of Fe^3+^ increased rapidly again, which corresponded to a simultaneous increase in biogas production.

The concentration variation of Fe^2+^ was similar to that of Fe^3+^. Fe^2+^ in the digestive system can increase the growth and metabolism of microorganisms [54]. Fe^2+^ can also promote the acetic acid methanogenic pathway through the following Equation (1) [37]. More importantly, MBC as an electron transfer carrier provided conditions for the formation of DIET between *Methanococcus* and acetic acid oxidizing bacteria, and then *Methanococcus* used CO_2_ produced during the CH_3_COOH oxidation process to generate methane [55,56].

(1) $${\mathrm{Fe}}^{2+}+2{\mathrm{CH}}_{3}{\mathrm{COO}}^{-}+H_{2}O\to \mathrm{Fe}\left(\mathrm{OH}\right)\left({\mathrm{CH}}_{3}\mathrm{COO}\right)+{\mathrm{CH}}_{3}\mathrm{COOH}$$

## 4. Conclusions

In this study, after adding MBC, the low dosages at 5–40 mg/g TS showed the best biogas production performance and biodegradability, and the optimum dosage of MBC was 20 mg/g TS. Kinetic analysis showed that the Modified Gompertz Model was better than the Cone Model to simulate the biogas production process of sludge AD after adding MBC. The mechanism analysis showed that MBC promoted the AD process of sewage sludge mainly by providing continuous iron ions to the system, promoting the acetic acid methanogenic pathway. The potential ability to create a favorable digestion environment will open new doors for better understanding and application of magnetic biochar using COS as a raw material in a sludge mesophilic AD system. However, the limitation of this research is that only a batch experimental was considered. In the future, research should explore the effects of MBC in a long-term semi-continuous experiment. Additionally, further research is required to determine whether these lab-scale experiments can be scaled up and implemented on a factory scale.

## Figures and Tables

**Table 1:** Main physicochemical characteristics of sludge in this study.

Characteristics	Feed	Seed
Total solids (TS, g L^−1^ substrate)	79.60 ± 1.36	19.60 ± 0.44
Volatile solids (VS, g L^−1^ substrate)	46.50 ± 0.61	7.93 ± 0.21
Moisture (%)	92.12 ± 0.13	98.07 ± 0.04
pH	7.14 ± 0.03	7.05 ± 0.01
Soluble chemical oxygen demand (sCOD, mg L^−1^)	6257.43 ± 101.38	463.37 ± 29.45
Total polysaccharides (mg L^−1^)	413.86 ± 16.28	46.62 ± 3.20
Protein (mg L^−1)^	452.70 ± 5.22	37.43 ± 8.11
VFAs (mg L^−1^)	1684.90 ± 15.81	38.39 ± 7.35
NH4^+^_-_N (mg L^−1^)	1000.31 ± 3.04	681.50 ± 9.62

**Table 2:** Models used to describe the kinetic study of biogas production.

Model	Equation	Parameters
Modified Gompertz Model	$B_{t}=B_{0}exp\left\{-exp\left[\frac{R_{m}*e}{B_{0}}*\left(\lambda -t\right)+1\right]\right\}$, *t* ≥ 0	*R_m_*, *B*_0_, *λ*
Cone Model	$B_{t}=\frac{B_{0}}{1+{\left(k*t\right)}^{-n}}$, *t* > 0	*B*_0_, *k*, *n*

*B_t_*: cumulative biogas yield (mL g^−1^ VS_added_); *B*_0_: potential biogas production (mL g^−1^ VS_added_); *R_m_*: maximum biogas production rate (mL g^−1^ VS d^−1^); *λ*: lag phase time (d); *t*: AD time (d); *k*: hydrolysis rate constant (d^−1^); *n*: shape factor; *e* ≈ 2.7183.

**Table 3:** Kinetic parameters of AD simulated by the Modified Gompertz Model.

Reactors	*B*_0_ (mL g^−1^ VS_added_)	*R_m_*	*λ*	R^2^	Measured (mL g^−1^ VS_added_)	Difference (%)
CK	311.10	32.23	2.01	0.9988	309.20 ± 4.59	0.61
MB_1	354.48	33.37	1.46	0.9997	353.58 ± 2.31	0.26
MB_2	373.25	34.24	1.27	0.9995	372.28 ± 1.23	0.26
MB_3	401.18	36.10	1.11	0.9994	400.16 ± 4.22	0.25
MB_4	440.77	39.18	1.02	0.9995	439.71 ± 1.97	0.24
MB_5	420.57	33.66	1.91	0.9993	418.54 ± 1.73	0.49

**Table 4:** Kinetic parameters of AD simulated by the Cone Model.

Reactors	*B*_0_ (mL g^−1^ VS_added_)	*K*	*n*	R^2^	Measured (mL g^−1^ VS_added_)	Difference (%)
CK	316..47	0.1470	2.83	0.9941	309.20 ± 4.59	2.35
MB_1	364.12	0.1491	2.45	0.9961	353.58 ± 2.31	2.98
MB_2	384.55	0.1504	2.35	0.9956	372.28 ± 1.23	3.30
MB_3	414.46	0.1517	2.27	0.9958	400.16 ± 4.22	3.57
MB_4	456.13	0.1522	2.23	0.9957	439.71 ± 1.97	3.73
MB_5	435.78	0.1227	2.43	0.9971	418.54 ± 1.73	4.12

## Data Availability

Data sharing is not applicable to this article.
